# Interactions between Affective and Cognitive Processing Systems in Problematic Gamblers: A Functional Connectivity Study

**DOI:** 10.1371/journal.pone.0049923

**Published:** 2012-11-28

**Authors:** Ruth J. van Holst, Johan N. van der Meer, Donald G. McLaren, Wim van den Brink, Dick J. Veltman, Anna E. Goudriaan

**Affiliations:** 1 Academic Medical Center, Department of Psychiatry, University of Amsterdam, Amsterdam, The Netherlands; 2 Amsterdam Institute for Addiction Research, Amsterdam, The Netherlands; 3 Massachusetts General Hospital, Department of Neurology and Athinoula A. Martinos Center for Biomedical Imaging, Department of Radiology, Boston, Massachusetts, United States of America; 4 Department of Psychiatry, VU University Medical Center, Amsterdam, The Netherlands; 5 Arkin Mental Health Care, Amsterdam, The Netherlands; 6 Edith Nourse Rogers Memorial Veterans Affairs Medical Center, Geriatric Research Education and Clinical Center, Bedford, Massachusetts, United States of America; 7 Harvard Medical School, Boston, Massachusetts, United States of America; George Mason University/Krasnow Institute for Advanced Study, United States of America

## Abstract

**Background:**

Motivational and cognitive abnormalities are frequently reported in pathological gambling. However, studies simultaneously investigating motivational and cognitive processing in problematic gamblers are lacking, limiting our understanding of the interplay between these systems in problematic gambling. Studies in non-clinical samples indicate that interactions between dorsal “executive” and ventral “affective” processing systems are necessary for adequate responses in various emotive situations.

**Methods:**

We conducted a generalized Psycho-Physiological Interaction (gPPI) analysis to assess the influence of affective stimuli on changes in functional connectivity associated with response inhibition in 16 treatment seeking problematic gamblers (PRGs) and 15 healthy controls (HCs) using an affective Go-NoGo fMRI paradigm including neutral, gambling-related, positive and negative pictures as neutral and affective conditions.

**Results:**

Across groups, task performance accuracy during neutral inhibition trials was positively correlated with functional connectivity between the left caudate and the right middle frontal cortex. During inhibition in the gambling condition, only in PRGs accuracy of task performance was positively correlated with functional connectivity within sub-regions of the dorsal executive system. Group interactions showed that during neutral inhibition, HCs exhibited greater functional connectivity between the left caudate and occipital cortex than PRGs. In contrast, during inhibition in the positive condition, PRGs compared to HCs showed greater functional connectivity between the left caudate and occipital cortex. During inhibition trials in the negative condition, a stronger functional connectivity between the left caudate and the right anterior cingulate cortex in PRGs compared to HCs was present. There were no group interactions during inhibition in the gambling condition.

**Conclusions:**

During gamble inhibition PRGs seem to benefit more from functional connectivity within the dorsal executive system than HCs, because task accuracy in this condition in PRGs is positively correlated with functional connectivity, although the groups show similar connectivity patterns during gamble inhibition. Greater functional connectivity between the ventral affective system and the dorsal executive system in PRGs in the affective conditions compared to HCs, suggests facilitation of the dorsal executive system when affective stimuli are present specifically in PRGs.

## Introduction

Pathological gambling, currently classified as an impulse control disorder in the DSM-IV, has been regarded as a ‘behavioral addiction’ by many researchers [1], [2], [3] and is expected to be incorporated in the new DSM-V under the new category of “Addiction and Related Disorders”. The disorder is characterized by loss of control over gambling behavior and continuation of gambling regardless of negative consequences. Despite the phenomenological evidence of abnormalities in a variety of cognitive and motivational functions in problem gambling and its neural mechanisms [4], studies addressing both functional systems simultaneously are lacking. This limits our understanding of the interplay between these systems in problem gambling, which is needed to further elucidate the etiology of this disorder.

Adequate behavior requires continuous coordination between initiation and inhibition of actions, the latter being particularly important when sudden changes in the situation call for a cancellation of planned or ongoing behavior. The cognitive executive process of response inhibition is responsible for interruption of ongoing behavior and depends on the right inferior frontal cortex (IFC; especially the frontal operculum extending into the insula), the superior frontal cortex (SFC) and the medial frontal cortex (MFC; particularly the pre-supplementary motor areas) [5], [6], [7], [8], [9], [10]. Evidence from functional magnetic resonance imaging (fMRI) studies in healthy adults indicates that affective information has a regulatory role in goal directed behavior through reciprocal interactions between *dorsal “executive”* and *ventral “affective” processing systems* [11], [12], [13], [14]. Several studies have revealed that this interaction between prefrontal cognitive control regions and limbic affective processing areas is critically involved in regulating attention and response selection in the presence of affective information [11], [13], [15], [16].

In addictive disorders, including pathological gambling, there is evidence that both affective and motivational systems are more sensitive to addiction relevant material. For example, studies have shown that addiction related cues attract more attention than other salient stimuli, a phenomenon known as “attentional bias” [17], [18], [19]. In problematic gamblers, enhanced brain responsiveness towards gambling related cues (“cue reactivity”) has also been found in brain areas related to motivational processing and cognitive control (amygdala, basal ganglia, ventrolateral prefrontal cortex and dorsolateral prefrontal cortex) [20], [21]. The *incentive sensitization* theory introduced by Robinson and Berridge [22], [23] explains attentional bias and cue reactivity as the result of sensitization of the mesocorticolimbic system following repeated exposure to addictive stimuli, associated with incentive salience to reward-associated stimuli and drug wanting. In addition, diminished executive functions such as disadvantageous choice behavior and diminished response inhibition have been reported in problem gamblers [24], [25], [26], [27], and has been associated with an attenuated BOLD response in the ventrolateral prefrontal cortex in problem gamblers compared to controls [28], [29], [30]. However, it is unclear whether the nature and extent of interactions between the *ventral “affective”* and *dorsal “executive” processing systems* in problematic gamblers differ from those in healthy controls.

In a previous fMRI study [31], we investigated the influence of affective stimuli (positive, negative and gambling related pictures) on response inhibition in problematic gamblers (PRGs) and healthy controls (HCs) during an affective Go-NoGo task. When presented with neutral pictures, response inhibition in PRGs was associated with more DLPFC and ACC activation, similar accuracy and slower reaction times compared to HCs. Stronger activation of DLPFC and ACC in combination with slower reaction times suggested a compensatory response and higher effort in PRGs to achieve the same accuracy as HCs. Interestingly, when an affective condition was introduced in the Go-NoGo task, PRGs were more accurate than HCs at response inhibition when confronted with gambling related and positive pictures and showed less activation of the relevant brain circuits, whereas negative pictures led to better task performance in both groups.

The facilitation of inhibition in PRGs compared to HCs when confronted with gambling and positive stimuli could be interpreted within the “dual process and competition” framework regarding the interaction between motivational and cognitive functioning [32], [33]. This model posits that affective stimuli influence competition for cognitive resources both at a perceptual and executive level. Thus, salience of affective stimuli will result in extra attention. This may facilitate task performance, such as discrimination or response inhibition tasks, but salient stimuli may also become overwhelming, and result in an overload of attentional resources and diminished cognitive control [32]. The finding that gambling related and positive pictures facilitated task performance more in PRGs than HCs indicates that increased attention towards these stimuli may have facilitated attentional network processing in PRGs compared to HCs.

From these results, it becomes clear that the interaction between cognitive and motivational brain areas may be crucial for a better understanding of the influence of salient stimuli on (the neural mechanisms of) cognitive control in PRGs. In this report, we present a new analysis of previously published fMRI data [31] using a functional connectivity technique, generalized Psycho-Physiological Interactions (gPPI; [34]), which allows us to investigate the effect of affective stimuli on functional connectivity patterns during response inhibition in PRGs and HCs. Two relevant seed regions were chosen: (1) the right inferior frontal cortex (rIFC) for its crucial role in response inhibition [5], [7], [35], [36], and (2) the left caudate for its role in the coding of affectively relevant stimuli [37], [38], [39]. We decided to use the term functional connectivity instead of effective connectivity [40] because PPI cannot be used to infer the directionality of the connection, so that we cannot state that the caudate/IFC affects other regions and vice versa.

First, we tested the general hypothesis that increased connectivity between the sub-regions of the dorsal executive system is associated with higher task accuracy, i.e., adequate response inhibition in both PRGs and HCs. This hypothesis is based on previous research showing a positive relation between task performance and functional connectivity with the task related network [36], [41], [42], [43], [44], [45]. For example, in a study on response inhibition using a stop signal task, psychophysiological interaction analyses showed that, successful stops evoked greater effective connectivity between the IFC and pre-supplementary motor areas than stop errors [36]. Therefore we hypothesized that better task accuracy, i.e. better response inhibition, would be related to higher connectivity within the dorsal frontal system. Second, we tested the hypothesis that in the neutral condition, functional connectivity between the right IFC and other sub-regions of the dorsal executive system is stronger in HCs than in PRGs, based upon our previous findings of more efficient task performance in HCs compared to PRGs [31]. Given the findings of enhanced activation of the reward and motivational brain system in gamblers toward gambling stimuli [20], [21], our third hypothesis was that gambling related stimuli will enhance functional connectivity between the ventral affective and the dorsal executive systems during response inhibition more in PRGs than in HCs. Finally, we explored group by condition interaction effects and the modulatory effect of positive and negative affective stimuli on functional connectivity during inhibition trials in PRGs and HCs.

## Methods

### 2.1 Subjects

A total of 16 male problematic gamblers (PRGs) and 15 male healthy controls (HCs), all right-handed, participated in this study. PRGs were recruited from Dutch addiction treatment centers where they received cognitive behavioral therapy. HCs were recruited through advertisements in local newspapers. Because most treatment-seeking PRGs are men, only male participants were included in the study. The main inclusion criterion for PRGs was current treatment for gambling problems. PRGs were interviewed with section T of the Diagnostic Interview Schedule [46] to assess the diagnostic criteria for a DSM-IV-TR diagnosis of pathological gambling. In addition, the South Oaks Gambling Screen (SOGS) [47] was administered, as a general indication of the severity of gambling problems and to facilitate comparisons with other studies using the SOGS.

Exclusion criteria for both groups were: lifetime diagnosis of schizophrenia or psychotic episodes; diagnosis of manic disorder (CIDI, section F), obsessive compulsive disorder (CIDI, section E), alcohol use disorders (CIDI, section J), substance dependence disorder (except for nicotine dependence) (CIDI, section L) or post-traumatic stress disorder (CIDI, section K); treatment for mental disorders other than pathological gambling in the past 12 months; use of psychotropic medication; difficulty reading Dutch; age under 18 years; positive urine screen for alcohol, amphetamines, benzodiazepines, opioids or cocaine; history or current treatment for neurological disorders, major internal disorders, brain trauma, or exposure to neurotoxic factors. In addition, HCs were excluded if they gambled more than twice a year. Subjects with a diagnosis of anxiety and/or depression were not excluded because of the considerable comorbidity between gambling and these disorders [48]. To obtain a measure of subjects’ global information processing speed, we administered the subscales Digit span and Number-Letter sequencing from the Wechsler Adult Intelligence Scale-Revised (WAIS-R) and combined these in a composite score for information processing speed [49].

The ethical review board of the Academic Medical Center approved the study and written informed consent was obtained from all subjects. Participants were reimbursed with 50 Euros transferred to their bank account following participation.

### 2.2 Paradigm

In order to test inhibition in the context of neutral and affective pictures we designed a Go-NoGo task that consisted of four blocks containing pictures that were positive, negative, neutral, or gambling-related. The paradigm ran on E-prime (Version 1.1. Pittsburgh, PA: Psychology Software Tools; 2004.) The positive, negative, and neutral pictures were selected from the International Affective Picture System (IAPS) [50] based on their valence and arousal scores. While positive pictures (mean: 7.6, SD 1.5) were higher in valence than neutral (mean: 5.3, SD 3.5) and negative pictures (mean: 2.4, SD 1.5), there were no differences in arousal scores between the positive and negative pictures (positive mean: 5.6, SD 2.1, negative mean: 5.2, SD 2.2, neutral mean: 3.5, SD 2.0) [50]. Gambling related pictures were taken from casino scenes, previously used in a study by Goudriaan et al. [21]. Pictures in each block were matched on visual properties such as brightness and complexity.

**Figure 1:**
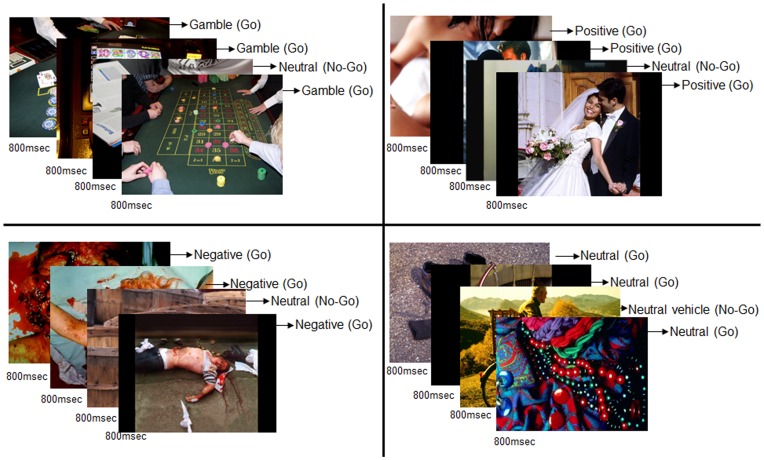
Example of the Go/Nogo task. Participants had to respond to affective pictures and try to withhold a response to neutral pictures.

Before each block started, an instruction appeared on the screen for 15 seconds, instructing participants to press a button when a certain type of stimulus was shown (Go trials) and to inhibit pressing the button when a neutral stimulus type was shown (NoGo trials). Each block consisted of 35 pictures, which were shown 4 times, presented in rapid succession for 800 ms each, thus each block had a duration of 112 seconds. To evoke an automated response, 100 Go trials and 40 NoGo trials were presented. NoGo trials never occurred more than twice in a row. In the gambling block, for example, the instruction was to respond as accurately and fast as possible to gambling-related pictures, and not to respond to neutral pictures (see Figure 1). Because all pictures were neutral in the neutral block, participants were instructed to respond to all neutral pictures, but not to respond when a vehicle was shown in the picture (40 of the 140 trials). An 8-item gambling urge questionnaire, with answer categories ranging from 1 (do not agree) to 7 (very much agree) [40] was included to assess the degree of craving for gambling. All subjects completed this urge questionnaire before and immediately after the gamble condition during fMRI scanning.

Behavioral outcomes of interest included accuracy (inverse of the percentage of impulsive errors, i.e. responding to NoGo trials) and mean reaction times in the different blocks.

### 2.3 Imaging Acquisition and Preprocessing

Imaging data were obtained using a 3.0 Tesla Intera full-body fMRI scanner (Philips Medical Systems, Best, The Netherlands) with a phased array SENSE RF eight-channel receiver head coil. 35 axial slices (voxel size 2.29×2.29×3 mm, no interslice gap, matrix size 96×96, field-of-view [FOV] = 220×220 mm, repetition time [TR] = 2.3 sec, echo time [TE] = 30 ms, flip angle = 80°, bandwidth 90 kHz) of T2*-weighted echo planar images (EPIs), sensitive to blood oxygenation level-dependent (BOLD) contrast were obtained, covering the entire brain except for the inferior regions of the cerebellum. A T1-weighed structural scan (T1 turbo field echo, TR = 9.6 seconds, TE = 4.6 ms, 182 sagittal slices, slice thickness 1.2 mm, FOV 256×256 mm, in-plane resolution 256×256, flip angle = 8°) was collected for coregistration with the fMRI data. Imaging analysis was performed using SPM5 (Statistical Parametric Mapping; Wellcome Trust Centre for Neuroimaging, London, UK). Images were manually reoriented and subsequently slice-time corrected, realigned and unwarped using automated procedures provided by SPM5. Next, registration of the T1-scan to the mean image, warping to Montreal Neurological Institute (MNI) space as defined by the SPM5 T1-template, reslicing to 3×3×3 mm voxels and spatial smoothing using an 8-mm FWHM Gaussian kernel was performed. Subjects with head movement over 3 mm in more than one direction were excluded from the analysis.

### 2.4 Statistical Analysis

To enable comparisons with our paper on this modified Go-NoGo task using conventional fMRI general linear model (GLM) analyses [31] we pre-processed the data in an identical way. The main group results of these analyses were used for the selection of the coordinates of the seed regions for the gPPI analyses.

All fMRI data were analyzed within the context of the General Linear Model, using delta functions convolved with a canonical hemodynamic response function to model responses to each type of stimulus that was correctly responded to [(affective block × Go/NoGo) resulting in 8 regressors], 1 regressor for incorrect Go trials, 1 regressor for incorrect NoGo trials, 1 regressor for introduction of a new condition and 1 regressor for craving questions (which indicated the onset and duration of the introduction blocks and craving questions, respectively, and which were included as nuisance regressors). Contrast images containing Go-NoGo parameter estimates were entered into a second-level (random effects) analysis.

Sociodemographic data were analyzed using univariate analysis of variance (ANOVA). Individual mean reaction times were based solely on correct responses. Reaction time data were tested for differences between groups, conditions and group by condition interactions with repeated measures ANOVAs with condition as within subject effect. This was followed up by separate ANOVAs to test group differences on the separate conditions. Non-normally distributed data (i.e. SOGS, craving scores, percentage of errors) were analyzed using Mann-Whitney U-tests for the comparison between groups. Friedman’s ANOVAs were used to test differences between experimental conditions within groups (percentage of errors during the different blocks) followed up by Wilcoxon tests for post-hoc comparisons. All analyses were performed two-tailed with an alpha level of 0.05.

### 2.5 Generalized Psycho-Physiological Interaction (gPPI) Seed Regions

**Figure 2:**
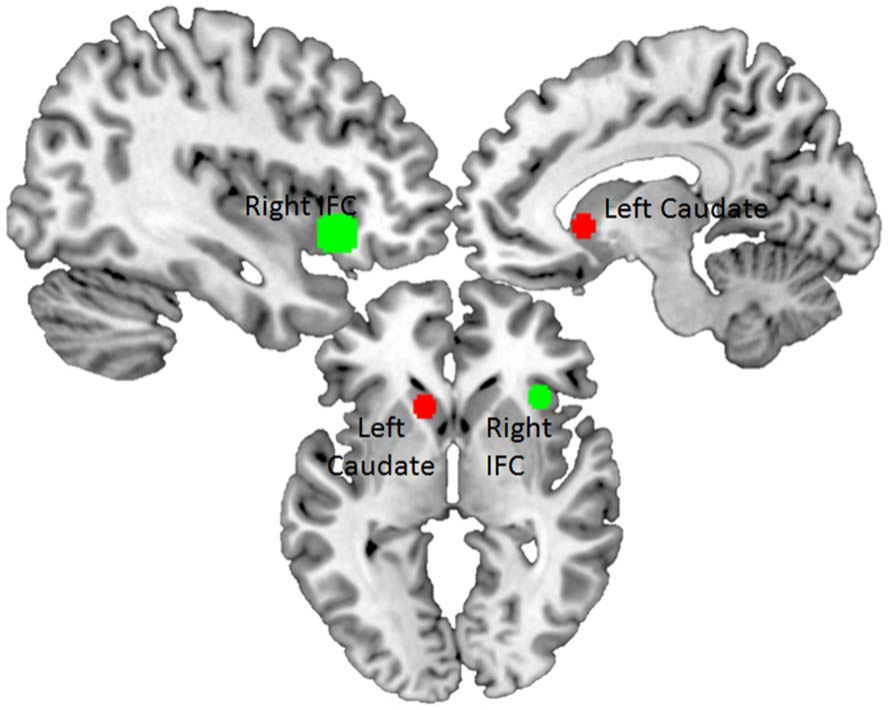
Location of seed regions. The seed regions were defined as 5- (caudate) and 8-mm (inferior frontal cortex) radius spheres with the origin at specific coordinates based on the group-analysis results. In green, the right inferior frontal cortex (MNI coordinates: 36, 21, −9). In red, the left caudate (MNI coordinates: −12, 24, −3). R  =  right hemisphere, L  =  left hemisphere.

Seed regions were chosen based on their involvement in response inhibition and affective processing. Seed regions were defined as radius spheres with the origin at specific coordinates based on the group-analysis results of the General Linear model [31]. In order to select a seed region for the dorsal executive system, we tested the activation across groups for the contrast Neutral NoGo> Neutral Go (p<0.001, uncorrected) because this contrast is the least likely to be confounded by affective processing. Please see Table S1 for all brain regions related to this ‘response inhibition’ contrast. Based on the peak voxels taken from this contrast, the best seed region for response inhibition was the right inferior frontal cortex (rIFC; MNI-coordinates: 36, 21, −9, with an 8 mm radius sphere). The ventral affective system related seed region was derived from the combined contrasts of Gambling Go>Neutral Go, Positive Go>Neutral Go, and Negative Go>Neutral Go (p<0.001, uncorrected). We used this contrast to make sure that we captured all affective processing activity. Please see Table S1 for all brain regions related to this affective response contrast. Based on the peak voxels taken from this combined contrast, the best seed region for affective processing was the left caudate (MNI-coordinates: −12, 24, −3, with a 5 mm radius sphere) (Figure 2).

### 2.6 Generalized Psychophysiological Interaction Analyses

We used generalized PPI (gPPI; https://www.nitrc.org/projects/gppi) [34], which has the flexibility to accommodate more than two task conditions in the same PPI model and is briefly described below.

For each subject and for each seed region, the physiological activity of the seed regions was computed as the mean time series of all voxels within an 5 or 8 mm radius sphere, depending on the left caudate or right IFC, respectively, centered at the aforementioned peaks from the group analyses (Figure 2). An estimate of the underlying neuronal activity that produced the physiological activity in the seed region was computed by deconvolving the BOLD signal [51]. Next, the 12 psychological/task vectors used in our PPI analysis included 8 affective blocks × Go/NoGo, 1 regressor for incorrect Go trials, 1 regressor for incorrect NoGo trials, 1 regressor for introduction of a new condition and 1 regressor for craving questions, were each multiplied by the estimated neuronal activity from the seed region and convolved with the canonical HRF. The 12 vectors were also convolved with the canonical HRF to form the psychological/task regressors. Then, a whole-brain analysis (single-subject level) was performed using the general linear model in SPM8 with the 12 PPI regressors, 12 psychological/task regressors and the mean time course in the seed region.

For each seed region, 8 PPI contrasts were created: neutral NoGo >baseline, gamble NoGo>neutral NoGo, positive NoGo >neutral NoGo, negative NoGo >neutral NoGo, gamble NoGo, positive NoGo and negative NoGo. The first contrast (neutral NoGo>baseline) identified regions having a functional connectivity between the seed region and other regions in the brain. The preceding contrasts (affective NoGo>neutral NoGo) identified functional connectivity changes of the seed region with other regions in the brain for affective inhibition (i.e. gamble inhibition, positive inhibition and negative inhibition compared to neutral inhibition). For each seed region, these individual PPI contrast images were entered into a two-sample *t*-test at the second (group) level to test between group differences. Group by condition interaction effects were tested with a full factorial design including the contrast gambling NoGo, positive NoGo, and negative NoGo.

Separate multiple regression analyses were performed on the PPI contrast images of *neutral inhibition, gamble inhibition, positive inhibition,* and *negative inhibition,* acquired with the right IFC and left caudate as seed regions and with task performance (percentage of errors during the different blocks) and group membership as covariates.

**Figure 3:**
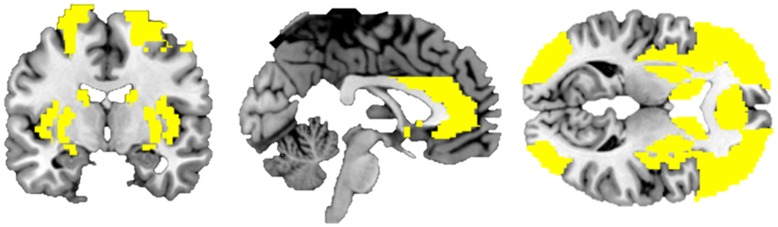
AAL mask of all combined regions of interest (ROIs) used for small volume correction analyses. We defined the inferior frontal cortex (IFC), anterior cingulate cortex (ACC), middle frontal cortex (MFC) and superior frontal cortex (SFC), the amygdala, caudate nucleus, putamen, insula, and occipital cortex as regions of interest (ROIs). All ROIs were defined using the WFU PickAtlas Tool v2.4 [12] which incorporates the automatic anatomical labeling (AAL) atlas [13] and were included in one mask holding all the ROIs.

All analyses were performed using a-priori regions of interest (ROIs) (Figure 3). We defined the inferior frontal cortex (IFC), anterior cingulate cortex (ACC), middle frontal cortex (MFC) and superior frontal cortex (SFC) as ROIs given their role in response inhibition [3]–[8]. The amygdala, caudate nucleus, putamen, insula, and occipital cortex were selected because of their involvement in the processing of affective information [eg., 9;10;11]. All ROIs were defined using the WFU PickAtlas Tool v2.4 [12], which incorporates the automatic anatomical labeling (AAL) atlas [13], and all ROIs were simultaneously included in one mask. Using the peak_nii toolbox (http://www.nitrc.org/projects/peak_nii), statistical images were thresholded at a multiple comparison corrected level of cluster FDR p<0.05 using small-volume correction within the aforementioned mask.

## Results

### 3.1 Demographical and Clinical Characteristics

Demographical and clinical characteristics are presented in detail in Table 1. There was no significant difference between the groups in terms of age and general cognitive performance (total score on the subscales Digit span and Number-Letter sequencing from the Wechsler Adult Intelligence Scale-Revised) [49]. As expected, PRGs had higher SOGS scores than HCs and all of them fulfilled the criteria for ‘probable pathological gambler’ defined by a SOGS score of five or more. Furthermore, except for one PRG, all PRGs met criteria of a current DSM-IV-TR pathological gambling diagnosis. Whereas none of the HCs met the diagnosis for depression and/or anxiety disorder, four PRGs met the diagnosis for major depression and one for generalized anxiety disorder. Before scanning, PRGs had significantly higher gambling craving scores than HCs. However, after performing the gamble block, gambling craving scores were increased in both groups (for HCs: (χ^2^(1) = 8.07, p<0.005; and for PRGs: (χ^2^(1) = 4.57, p<0.03), and there was no group difference on gambling craving after the gamble block (see Table 1 for a summary of these data).

**Table 1:** Demographic and clinical information.

	HCs N = 15	PRGs N = 16	Significance (ANCOVA; Mann-Whitney U)
Age, mean (SE)	36.20 (10.69)	34.38 (11.14)	F(1,30) = 0.22 p = 0.65
WAIS composite score, mean (SE)	15.40 (1.02)	13.75 (0.71)	F(1,30) = 1.80 p = 0.19
SOGS*, mean (SE)	0.07 (0.26)	11.57 (3.00)	U = 0, p = 0.000
Gambling craving before task*, mean (SE)	8.27 (2.58)	16.56 (10.26)	U = 50, p = 0.005
Gambling craving after task, mean, (SE)	17.80 (13.06)	21.50 (11.63)	U = 87, p = 0.20

HCs = Healthy controls, PRGs = Problematic gamblers, WAIS composite score = composite score of the subscales Digit span and Number-Letter sequencing from the Wechsler Adult Intelligence Scale-Revised; SOGS  =  South Oaks Gambling Screen, SE  =  standard error;

* = significant group difference at p<0.05.

### 3.2 Behavioral Performance on the Go-NoGo Task

Significant main effects for condition (F(3,26) = 22.059, p = 0.001) and for group (F(1,29) = 8.075, p = 0.008) were present. PRGs responded slower than HCs (PRGs Mean = 500.36 msec, SE = 8.61 and HCs Mean = 465.19 msec, SE = 8.89). PRGs were significantly slower compared to HCs during the negative condition and during the positive condition, whereas a trend in the same direction was present for the neutral condition and for the gamble condition (see Table 2A).

**Table 2:** Reaction times and impulsive errors during neutral, gamble, positive, and negative conditions.

2A:	HCs		PRGs				
Reaction times in Msec	Mean	SE	Mean		SE		Statistics
Neutral Go trials*	486.15	9.40	515.58		11.43		F(1,30) = 3.90, p* = *0.058
Gambling Go trials*	455.52	7.66	481.69		11.09		F(1,30) = 3.68, p = 0.065)
Positive Go trials**	480.78	10.31	517.10		9.95		F(1,30) = 6.43, p* = *0.017
Negative Go trials**	438.32	10.08	487.04		10.32		F(1,30) = 11.36, p = 0.002
**2B:**	**HCs**			**PRGs**			
**Percentage of impulsive errors**	**Mean**	**SD**		**Mean**	**SD**	**Statistics**	
Neutral condition	19.67	2.21		18.75	2.15	U = 0.31, p = 0.58	
Gamble condition**	17.67	2.23		7.97	1.73	U = 41.05, p = 0.001	
Positive condition*	21.00	3.36		13.28	2.04	U = 73.50, p = 0.066	
Negative condition	13.00	1.68		12.03	2.01	U = 0.25, p = 0.62	

HCs  =  Healthy controls, PRGs  =  Problematic gamblers,

** = significant group difference at p<0.05;

* = trend for group differences *p*<0.10; SD  =  standard deviations; Error bars represent the standard deviations of the mean.

For impulsive errors we found a main effect of condition F(3,26) = 8.636, p = 0.001) and a group × condition interaction F(3,26) = 5.612, p = 0.006). Between group analyses indicated a trend for PRGs, who had a higher task accuracy compared to HCs (F(1,28) = 3.067, p = 0.068). Post-hoc analyses showed that PRGs made significantly less impulsive errors compared to HCs during the gamble condition, a trend in the same direction was present for the positive condition (see Table 2B).

A within-group repeated measures analysis showed a significant effect of stimulus condition on the percentage of impulsive errors in the HCs (χ2(3) = 8.69, p<0.034). Post-hoc analyses indicated that HCs performed best during the negative block compared to the other blocks (negative block compared to neutral block: T = 5, p<0.007, negative block compared to gamble block: T = 231, p<0.034, negative block compared to positive block: T = 7.5, p<0.008). Also in PRGs, a significant effect of condition on the percentage of impulsive errors was present (χ2(3) = 17.34, p<0.001). Here, post-hoc tests showed that PRGs performed best during the gamble block compared to the other blocks (gamble - neutral block: T = 6.5 p<0.001, gamble - positive block: T = 23.5 p<0.038, gamble - negative block: T = 9.5 p<0.020). Furthermore, PRGs made fewer impulsive errors during the positive and negative block compared to the neutral block (positive block compared to neutral block: T = 25, p<0.046, negative block compared to neutral block: T = 11, p<0.005). There was no performance difference between the positive and negative block in PRGs.

Results from the Spearman correlation analyses showed only one significant negative correlation, between the percentage of impulsive errors on the positive condition and reaction time (r = −0.379, N = 30, p = 0.030), indicating that in the positive condition, slower response times were associated with better task performance across groups. However, when testing the Spearman correlations in each group separately we found no significant correlations between the percentage of impulsive errors and reaction times.

### 3.3 Task Performance and Functional Connectivity

First, we tested the general hypothesis that greater connectivity between prefrontal dorsal cortical regions would be associated with task accuracy, i.e., better response inhibition in both PRGs and HC.

#### 3.3.1 Task performance and connectivity during neutral inhibition

**Figure 4:**
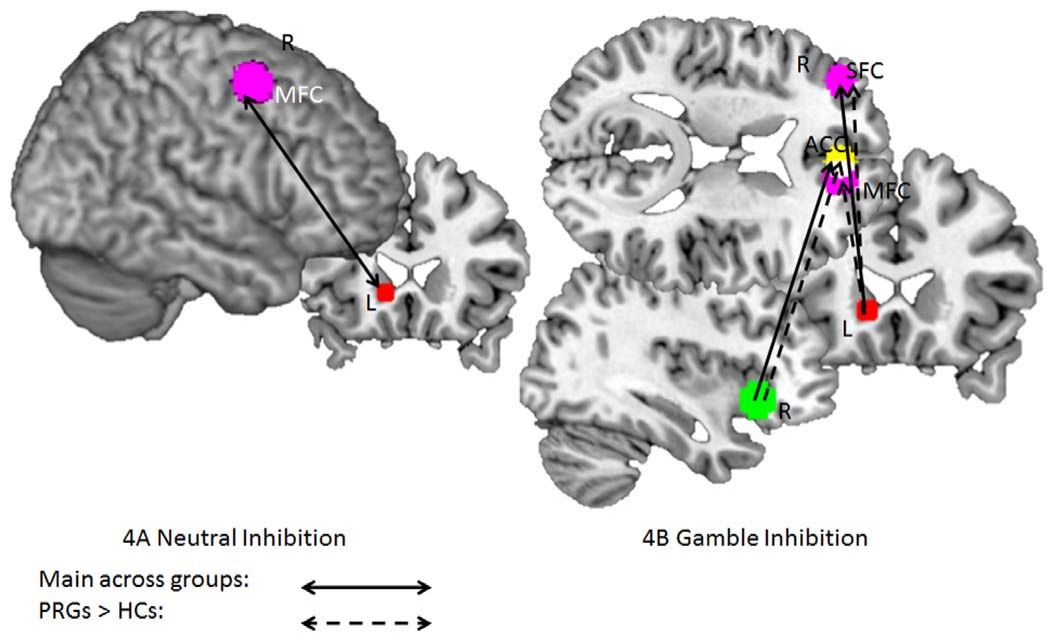
Schematic figure depicting functional connectivity patterns between brain regions showing a positive correlation with task performances during different conditions. HCs: healthy controls; PRGs: problematic gamblers; arrows = connectivity, R  =  right hemisphere; L  =  left hemisphere, green = seed region IFC, red = seed region caudate, yellow  =  connectivity with right IFC, violet = connectivity with left caudate; for specific peak voxel coordinates, see text (Results).

Regression analyses indicated that in the neutral condition, across groups, better task performance was associated with functional connectivity between the left caudate and the right MFC (15 voxels; MNI coordinates: 36, 3, 57; Z-value = 4.19; FDRsvc = 0.068), see Figure 4A. Connectivity between the right IFC and other regions was not significantly correlated with accuracy of task performance. There were no group differences in regression slope between functional connectivity and task performance.

#### 3.3.2 Task performance and connectivity during gamble inhibition

Better task performance during response inhibition when viewing gambling pictures across groups was associated with functional connectivity between the right IFC and the right ACC (25 voxels; MNI coordinates: 3, 45, 21; Z-value = 4.02; FDRsvc = 0.034) and between the left caudate and right SFC (68 voxels; MNI coordinates: 27, 60, 21; Z-value = 4.40; FDRsvc<0.001). However, this result was driven by a significant correlation within the PRGs group showing correlations between task accuracy and functional connectivity between the right IFC and the right ACC (21 voxels; MNI coordinates: 3, 45, 21; Z-value = 4.02; FDRsvc = 0.044) and between the left caudate and right SFC and left MFC (57 voxels; MNI coordinates: 27, 60, 21; Z-value = 4.11; FDRsvc<0.001 and 90 voxels; MNI coordinates: −33, 48,12; Z-value = 4.06; FDRsvc<0.001, respectively)_._ Compared to HCs, PRGs showed a stronger positive correlation between task accuracy and functional connectivity between the left caudate and bilateral MFC (49 voxels; MNI coordinates: 27, 60, 21; Z-value = 4.11; FDRsvc = 0.004 and 123 voxels; MNI coordinates: −36, 45, 9; Z-value = 4.08; FDRsvc<0.001, respectively), see Figure 4B. There were no significantly stronger correlations between task performance and functional connectivity for HCs compared to PRGs.

#### 3.3.3 Task performance and connectivity during positive inhibition

Better task performance during response inhibition when viewing positive pictures was not associated with functional connectivity between the right IFC and left caudate seeds. There were no group differences in regression slope between functional connectivity and task performance.

#### 3.3.4 Task performance and connectivity during negative inhibition

Better task performance during response inhibition with positive pictures was not associated with functional connectivity between the right IFC and left caudate. There were no group differences in regression slope between functional connectivity and task performance.

### 3.4 Group Differences in Inhibition Related Connectivity

#### 3.4.1 Neutral condition inhibition

**Figure 5:**
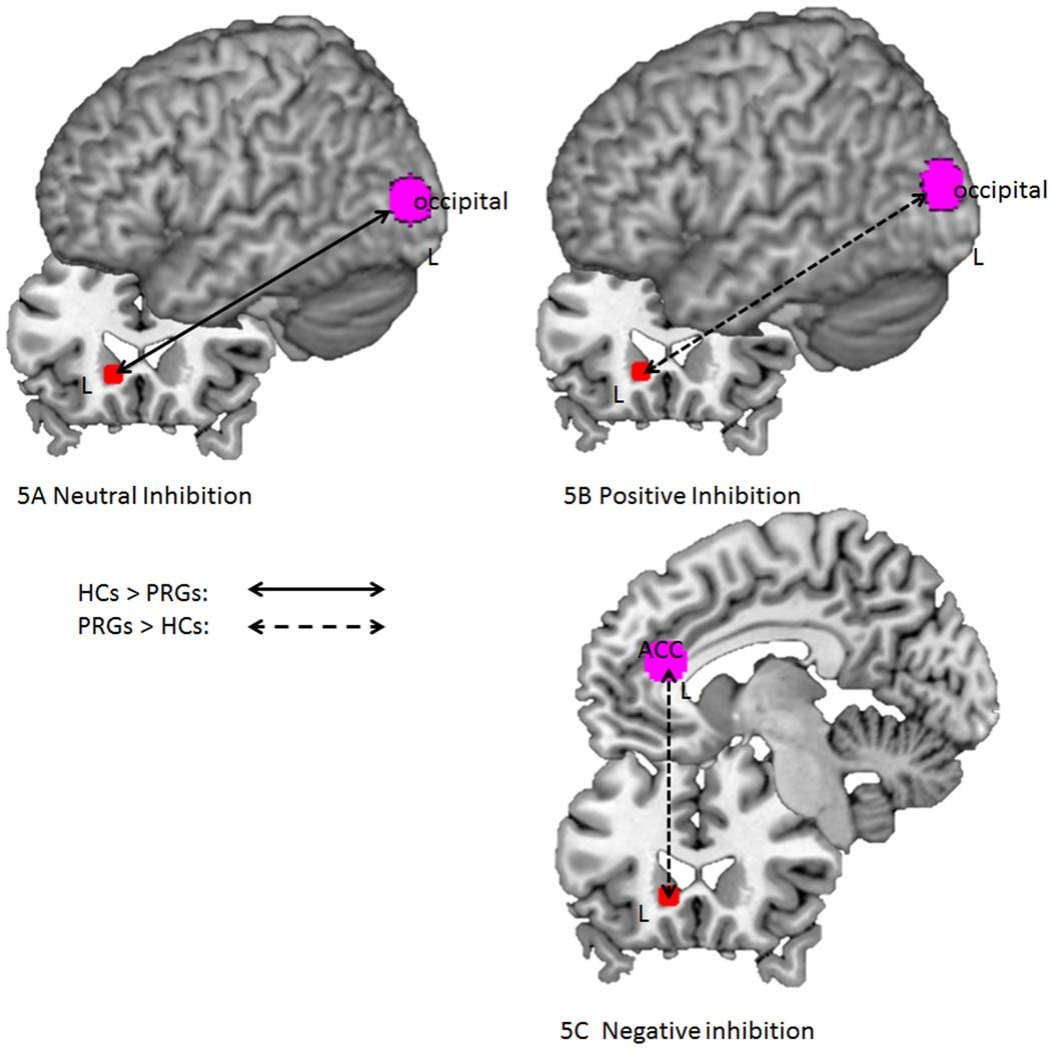
Schematic figure depicting functional connectivity patterns between brain regions during different conditions in HCs and PRGs. HCs: healthy controls; PRGs: problematic gamblers; arrows = connectivity, R  =  right hemisphere; L  =  left hemisphere, red = seed region caudate, violet = connectivity with left caudate; for specific peak voxel coordinates please see text (Results).

Inhibition during presentation of neutral stimuli was associated with greater functional connectivity between the left caudate and the left occipital cortex in HCs compared to PRGs (49 voxels; MNI coordinates: −33, −90, 9; Z-value = 4.75; FDRsvc = 0.002), see Figure 5A. There were no functional connectivity patterns that were greater for PRGs compared to HCs.

#### 3.4.2 Gamble condition inhibition (versus neutral inhibition)

There were no group differences in functional connectivity during inhibition in the Gamble condition.

#### 3.4.3 Positive condition inhibition (versus neutral inhibition)

The only group difference present indicated greater functional connectivity in PRGs compared to HCs between the left caudate and the left occipital cortex (26 voxels; MNI coordinates: −27, −90, 15; Z-value = 3.72; FDRsvc = 0.032). See Figure 5B.

#### 3.4.4 Negative condition inhibition (versus neutral inhibition)

Group comparisons indicated that PRGs showed greater functional connectivity between the left caudate and the left ACC compared to HCs (38 voxels; MNI coordinates: −3, 33, 18; Z-value = 4.47; FDRsvc = 0.026). See Figure 5C. There were no functional connectivity patterns that were greater for HCs compared to PRGs.

#### 3.4.5. Group*condition interaction effects

The contrasts gamble NoGo - positive NoGo and gamble NoGo - negative NoGo trials yielded no significant group differences in connectivity with the right IFC and the left caudate seed.

**Figure 6:**
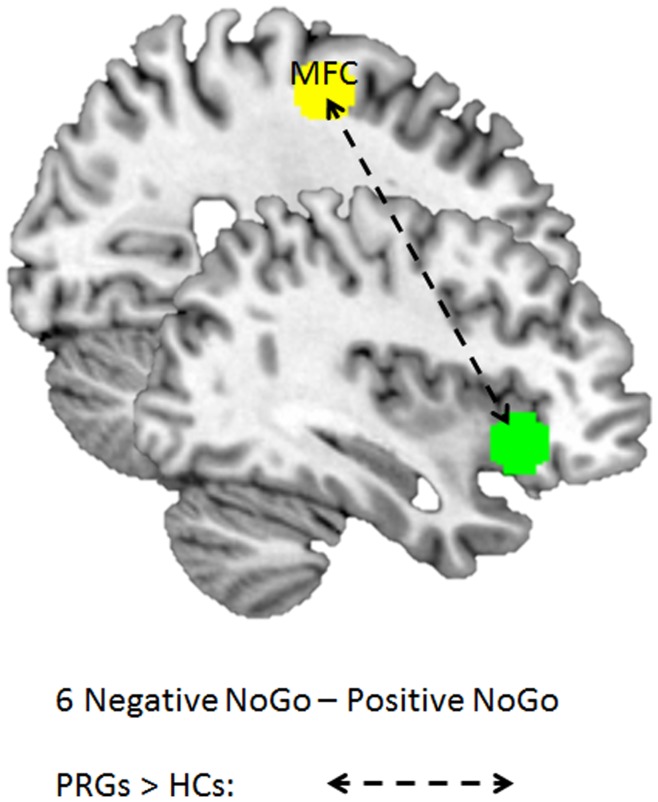
Schematic figure depicting functional connectivity patterns for group × picture interactions in HCs and PRGs. HCs: healthy controls; PRGs: problematic gamblers; arrows = connectivity, green = seed region right IFC, yellow  =  connectivity with right IFC, for specific peak voxel coordinates please see text (Results).

Negative NoGo – positive NoGo trials indicated more functional connectivity for PRGs compared to HCs between the right IFC and the right MFC (23 voxels; MNI coordinates: 30, 9, 54; Z-value = 4.02; FDRsvc = 0.012). See Figure 6. There were no functional connectivity patterns that were greater for HCs compared to PRGs.

## Discussion

The main goal of the current study was to investigate functional brain connectivity patterns between the motivational/affective system and the cognitive executive system in PRGs and HCs during a Go-NoGo task including neutral response inhibition, response inhibition when presented with gambling related pictures and positive and negative pictures. Furthermore, we tested the relationship between evoked functional connectivity during the various affective conditions and task performance in both groups.

On a behavioral level, PRGs showed similar accuracy but slower reaction times on neutral and negative trials compared to HCs. Previous studies on this issue have been inconsistent with some studies reporting diminished response inhibition in pathological gamblers and other studies failing to observe response inhibition impairments in pathological gamblers [4]. Interestingly, we did find behavioral differences on response inhibition depending on the affective stimuli presented in the Go-NoGo task. PRGs were more accurate than HCs at response inhibition when confronted with gambling related and positive pictures, whereas negative pictures were associated with better task performance in both groups.

### 4.1 Functional Connectivity within the Dorsal Cognitive Prefrontal System is Associated with Increased Accuracy of Response Inhibition

Based on previous studies [5], [6], [7], [8], [9], [10], we hypothesized that increased accuracy of response inhibition would be positively related to increased functional connectivity within the dorsal executive system. Our findings partly support this hypothesis; we found that during neutral inhibition trials task accuracy was positively associated with connectivity between the left caudate and the right MFC, which is part of the dorsal executive system. The absence of a positive correlation between task performance and functional connectivity in the positive and negative conditions was not in line with our hypothesis. However, we used a quite stringent threshold to correct for multiple comparisons, whereas when a more lenient threshold was applied, significant functional connectivity between the right IFC and other prefrontal cortex regions was revealed (data not shown).

### 4.2 Negative and Positive Affective Stimuli Evoke More Functional Connectivity in PRGs than in HCs

Although we expected that HCs compared to PRGs would show greater functional connectivity within the dorsal executive system during neutral inhibition representing more efficient task performance (i.e. faster and more accurate), we found that HCs had a stronger functional connectivity between the left caudate and the occipital cortex in the neutral condition. This suggests that, if anything, HCs applied more visual attention than PRGs, which could have led to more efficient performance in HCs. In the positive condition the opposite pattern was found; PRGs compared to HCs showed a stronger functional connectivity between the left caudate and the occipital cortex, which may indicate that positive affective pictures may increase motivation to perform and lead to higher attention to the task in PRGs compared to HCs. Although it seems likely that enhanced functional connectivity between the caudate and occipital cortex indicates enhanced visual attention [52] leading to better task performance [53], we did not find evidence of this positive correlation between this functional connectivity pattern and task performance, and we therefore have to interpret these functional connectivity differences between HCs and PRGs with caution.

Different from our hypothesis, we did not find any functional connectivity differences during gamble inhibition between the groups, although PRGs did show higher task accuracy than controls. Our regression analysis, however, showed that better task performance was more strongly correlated with functional connectivity between the right IFC and the SFC and MFC and between the left caudate and SFC and MFC in PRGs, but not in HCs. Thus, it seems that although functional connectivity patterns between groups were not different during gamble inhibition, task performance was more related to functional connectivity between the dorsal executive regions in PRGs compared to HCs.

During the negative inhibition trials we found that PRGs compared to HCs recruited more functional connectivity within the dorsal executive system (i.e. between right IFC and the right ACC). However, our regression analysis showed no positive correlation between functional connectivity within the dorsal executive system and task accuracy. This suggests that PRGs used a different strategy, involving more cognitive control regions, to perform similar to HCs on response inhibition when confronted with negative affective pictures. Because this study is the first to investigate the interaction between cognitive and motivational brain areas in pathological gambling, evidently more research is needed to elucidate the influence of salient stimuli on (the neural mechanisms of) cognitive control in PRGs.

### 4.3 Group*Condition Interaction

Group × condition interactions were only found with the contrast negative inhibition – positive inhibition, which indicated more connectivity within the dorsal system (IFC with MFC) for PRGs compared to HCs. These effects are probably best explained by the stronger connectivity pattern found in PRGs during negative inhibition compared to HCs. This stronger connectivity pattern found in PRGs when confronted with negative affective pictures suggests that PRGs are more sensitive to negative affective stimuli than HCs, which corresponds to findings of high anxiety and depression in pathological gamblers [48]. Indeed, studies in anxious and depressive cohorts have shown similar enhanced sensitivity to negative affective stimuli (e.g. attentional bias towards fearful pictures) [55]–[57]. Therefore, we conclude that compared to HCs, PRGs may be more sensitive to negative affective stimuli which facilitates attention and resources in the dorsal executive system.

### 4.4 Enhancement of Top-Down Control

Our finding that PRGs performed better on response inhibition during gambling related and positive conditions suggests that response inhibition can be facilitated by specific salient stimuli, associated with increased functional connectivity between the left caudate and the dorsal executive system. Salient stimuli may enhance transmission in the mesolimbic dopaminergic system [54], [55], [56], and dopamine is known to modulate prefrontal cortex functioning [57]. Indeed, in humans, altered dopamine transmission may affect functional connectivity within the cortico-striatal-thalamic loops [58], [59]. Only a few studies have directly investigated how dopamine modulates functional connectivity during a cognitive control task with use of fMRI. Of these, Nagano-Saito and colleagues [60] reported that participants with normal dopamine levels showed frontal-striatal functional connectivity that was positively related to faster response times during the Wisconsin Card Sorting Task. In addition, dopamine depletion in these participants resulted in impairment of frontal-striatal functional connectivity and less efficient task performance. This suggests that normal dopamine function supports both corticostriatal functional connectivity and efficient task performance. In the current study, during the neutral inhibition trials, PRGs showed less functional connectivity between the left caudate and the occipital cortex, which could be an indication of diminished visual attention towards neutral stimuli. However, it may be argued that in the present study salient (gambling-related and positive) stimuli, known to enhance DA transmission in the mesolimbic reward system [61], [62], could have transiently restored the hypoactive dopaminergic state in PRGs, facilitating normal functional connectivity between prefrontal brain regions during these conditions. Although this post-hoc explanation needs empirical testing, these findings seem relevant in the light of possible treatment targets for pathological gambling. Future research should further investigate whether increased activity in the reward system indeed has the effect to transiently restore prefrontal cortex functioning in PRGs, for example by pharmacological challenges or by enhancing activity in the reward system more locally, for example by using real time-fMRI neurofeedback [63], [64] or transcranial magnetic stimulation [65].

### 4.5 Strengths and Limitations and Suggestions for Future Research

This study has both strengths and limitations. Strengths include the fact that this is the first study showing that affective stimuli have a differential effect on functional connectivity patterns in PRGs and HCs and that this difference is associated with response inhibition performance. A limitation of the study is that we did not include subjective valence or salience ratings of the pictures by the participants themselves. However, we did select our pictures based on the IAPS valence and arousal ratings, which are well validated and tested in extensive samples [50]. In our study we chose to measure response inhibition in a task with infrequent neutral NoGo trials, while presenting our subjects with frequent affective Go pictures, and not to present them with infrequent affective NoGo trials and frequent neutral Go trials. The reason for choosing this design was that we expected that when the participants would see more affective Go pictures, this would elicit more cue-reactivity and craving, than when choosing the opposite design (neutral Go pictures, affective NoGo pictures). In future research, testing the full model, in order to examine response inhibition when confronted with addiction-related stimuli directly (i.e., presenting neutral go pictures and affective NoGo pictures) is advised. Thus, the hypothesis of reduced response inhibition in the face of addiction-related stimuli could be tested directly. Because of time constraints in the MRI scanner, we could not test this full model in the current study. Also, future research could benefit from including measures of personality traits related to appetitive motivation and approach behavior, because studies have shown that such traits affect participants’ behavior towards incentives [66], [67], [68], and may also modulate the effects of salient stimuli on brain activity [44], [66]. Notably, there is some evidence that PRGs reveal high scores on sensation seeking or reward seeking personality traits questionnaires [24], [69]. Future research should focus on how these personality traits are related to the function of the motivational system, and how this affects executive function in PRGs, to clarify the interaction of these factors in the etiology of PRGs.

### Conclusion

This study shows that adequate response inhibition is dependent on functional connectivity within the sub-regions of the dorsal executive system as well as on functional connectivity between the dorsal executive and the ventral affective system in both HCs and PRGs. Furthermore, in HCs neutral response inhibition is associated with increased functional connectivity between the left caudate and the occipital cortex. However, inhibition when confronted with positive stimuli result in enhanced functional connectivity in PRGs compared to controls between the left caudate and occipital cortex, whereas we did not find any group differences on functional connectivity during inhibition in the gambling condition. PRGs compared to HCs did show a stronger positive correlation between the dorsal executive system and task accuracy during inhibition in the gambling condition. Also, PRGs compared to HCs showed better response inhibition accuracy in the gamble and positive conditions. These findings could indicate that increased accuracy in PRGs during gambling and positive stimuli is associated with increased top-down control by the dorsal executive system in PRGs compared to HCs.

## Supporting Information

Table S1 **Seed region selection.** Seed regions were chosen based on their involvement in response inhibition and affective processing. Seed regions were defined as radius spheres with the origin at specific coordinates based on the group-analysis results of the General Linear model. Results are based on across group main effects tested with a whole brain voxel wise p<0.001 uncorrected. The bold and underlined regions are the corresponding selected seed regions.
(DOCX)
