# Supplementary material for: Interactions between Affective and Cognitive Processing Systems in Problematic Gamblers: A Functional Connectivity Study
Source: PLoS One. 2012 Nov 28;7(11):e49923. doi: 10.1371/journal.pone.0049923 (PMC3509135; doi:10.1371/journal.pone.0049923)
Supplement: Table S1 — Seed region selection. Seed regions were chosen based on their involvement in response inhibition and affective processing. Seed regions were defined as radius spheres with the origin at specific coordinates based on the group-analysis results of the General Linear model. Results are based on across group main effects tested with a whole brain voxel wise p<0.001 uncorrected. The bold and underlined regions are the corresponding selected seed regions. (DOCX) [file pone.0049923.s001.docx]

**Supplementary**

**Table S1**

**Main effect of neutral NoGo > neutral Go based on General Linear Model**

|  | **Voxels** | **R/L** | **x** | **y** | **z** | **Peak Z value** |
| --- | --- | --- | --- | --- | --- | --- |
| **Region** |  |  |  |  |  |  |
| Superior frontal | 6 | R | 18 | 27 | 57 | 3.26 |
| Medial superior frontal | 5 | R | 12 | 60 | 18 | 3.16 |
| Supplementary motor | 5 | R | 6 | -12 | 54 | 3.11 |
| Middle cingulum | 54 | R | 3 | -33 | 33 | 3.39 |
| **Inferior frontal cortex** | **16** | **R** | **36** | **21** | **-9** | **3.23** |
| Middle temporal | 318 | R | 48 | -36 | -3 | 4.38 |
|  | 21 | L | -45 | -48 | 15 | 3.16 |
| Inferior temporal | 7 | R | 45 | -72 | -9 | 3.12 |
| Precuneas | 55 | L | -12 | -54 | 54 |  |
| Supra marginal | 27 | L | -57 | -51 | 27 | 3.22 |
| angular | 26 | L | -54 | -60 | 39 | 3.27 |
| Occipital | 32 | L | -36 | -69 | 39 | 3.45 |
| Lingual | 23 | L | -12 | -63 | -6 | 3.17 |

**Main effect of Affective Go > neutral Go based on General Linear Model**

|  | **Voxels** | **R/L** | **x** | **y** | **z** | **Peak Z value** |
| --- | --- | --- | --- | --- | --- | --- |
| **Region** |  |  |  |  |  |  |
| Superior frontal | 291 | L | -12 | 48 | 45 | 4.68 |
| Post central | 7 | L | -45 | -9 | 36 | 3.66 |
| Inferior frontal | 6 | L | -42 | 30 | 3 | 3.61 |
| Anterior cingulate | 14 | L | -15 | 48 | 0 | 4.12 |
| Temporal pole | 37 | L | -36 | 9 | -30 | 4.14 |
| Middle temporal | 632 | R | 54 | -63 | 3 | 6.01 |
| **Caudate** | **25** | **L** | **-12** | **9** | **-3** | **3.79** |
| Precuneus | 520 | L | -9 | -54 | 33 | 5.47 |
| Occipital | 605 | L | -42 | -78 | -3 | 5.71 |
